# Booster vaccinations and Omicron: the effects on SARS-CoV-2 antibodies in Dutch blood donors

**DOI:** 10.1186/s12879-023-08448-w

**Published:** 2023-07-12

**Authors:** F. A. Quee, B. M. Hogema, E. Slot, S. Kruijer, M. Molier, K. van den Hurk, H. L. Zaaijer

**Affiliations:** 1 Department of Donor Medicine Research, Sanquin Research, Amsterdam, The Netherlands; 2 Department of Virology, Sanquin Diagnostic Services, Amsterdam, the Netherlands; 3 Department of Medical Affairs, Sanquin Corporate Staff, Amsterdam, the Netherlands; 4 Department of Experimental Immunohematology, Sanquin Research, Amsterdam, the Netherlands; 5 Department of Clinical Virology, Amsterdam University Medical Centre, Amsterdam, The Netherlands

**Keywords:** SARS-CoV-2, Antibodies, Blood donor, Vaccination, Antibody waning, Infection, Sero-surveillance

## Abstract

**Introduction:**

The severe acute respiratory syndrome coronavirus 2 (SARS-CoV-2) booster vaccination campaign and the emergence of SARS-CoV-2 Omicron variants impact the prevalence and levels of SARS-CoV-2 antibodies in the Netherlands. In this study we determined antibody levels across age groups, the impact of Omicron variant infections, and the effect of booster vaccinations on antibody levels.

**Methods:**

In September and December 2021 and in February 2022, over 2000 Dutch blood donors were tested for presence of SARS-CoV-2 antibodies. Donations were selected based on age, sex, and region of residence, to provide an optimal coverage and representation of the Dutch population.

**Results:**

Levels of vaccination-induced spike antibodies decreased over time in all age groups. Donors vaccinated with Janssen or AstraZeneca had significantly lower antibody levels than donors vaccinated with Pfizer or Moderna vaccine. Boostering with an mRNA vaccine elevated antibody levels in all age-groups irrespective of the initial vaccine. In donors aged < 56 years, the proportion of infected donors almost doubled between December 2021 and February 2022.

**Conclusion:**

The booster vaccination campaign increased antibody levels in all age-groups. After a booster vaccination, donors initially vaccinated with AstraZeneca or Janssen vaccine showed antibody levels similar to donors initially vaccinated with an mRNA vaccine. The emergence of the SARS-CoV-2 Omicron variant in the Netherlands caused a substantial increase in donors with infection-induced antibodies, especially among younger donors.

**Supplementary Information:**

The online version contains supplementary material available at 10.1186/s12879-023-08448-w.

## Introduction

Severe acute respiratory syndrome coronavirus 2 (SARS-CoV-2) is an infectious pathogen and the causative pathogen of corona virus disease 2019 (COVID-19) [1]. The disease was first documented in China, and spread rapidly across the world through person-to-person transmission [2]. The first case in the Netherlands was reported on February 27 2020, and was followed by a rapid increase in hospitalizations and deaths due to COVID-19 [3]. After the start of the vaccination campaign in January 2021, the amount of cases and hospitalizations decreased.

Starting October 2021, the number of severe acute respiratory syndrome coronavirus 2 (SARS-CoV-2) infections in the Netherlands rapidly increased again, due to the higher infectivity of the Delta variant (B.1.617.2) as compared to the Alpha variant (B1.1.7)[4, 5]. Because of the increasing number of infections and the waning of vaccine-induced antibody levels, a nationwide booster vaccination campaign started on November 18th 2021 [6]. Each fully vaccinated individual 18 years or older could receive a booster vaccination with one of two approved mRNA vaccines. At this point, approximately 84.4% of all Dutch adults was fully vaccinated [7]. On March 6th 2022, 86.4% of the population was fully vaccinated, and 62% had received a booster vaccination.

On November 26th 2021, a SARS-CoV-2 variant of concern was reported in South Africa, named Omicron (B.1.1.529) [8, 9]. Several days later the first Omicron infections were detected in the Netherlands, where it quickly became the dominant circulating variant, outcompeting the Delta variant. Due to mutations in the Spike (S) protein, Omicron is able to escape existing neutralizing antibodies, introduced by vaccination or previous infection [10, 11]. However, boostered individuals seemed to be better protected against severe disease after Omicron infection than non-boostered individuals [12].

To monitor effects of the booster vaccination campaign and the impact of Omicron on antibody responses, sero-surveillance is essential. It gives an estimation of the proportion of the population that has potentially protective antibodies against SARS-CoV-2, and it provides insight in the extent of the pandemic, long-term antibody trajectories, and the incidence of re- or breakthrough infections. By measuring antibodies to the receptor binding domain (RBD) of the spike protein, and antibodies to the nucleocapsid (NC) protein, differentiation is possible between vaccinated individuals, and infected individuals (with or without concomitant vaccination).

In this study, we cross-sectionally determined SARS-CoV-2 antibody levels in Dutch blood donors at three different timepoints. We aimed to determine (1) the extent of changes in mean antibody levels across age groups, in vaccinated persons and in (previously) infected persons, (2) the impact of the Omicron variant on the ratio between vaccinated and (previously) infected individuals, and (3) the effect of a booster vaccination on antibody levels.

## Methods

### Setting

#### The Dutch vaccination campaign and other preventive measures

On January 6th 2021 a nationwide SARS-CoV-2 vaccination campaign started in the Netherlands. People living in nursing homes, healthcare workers, and elderly were prioritized. Thereafter, starting at the highest age group, every citizen was invited to make an appointment for vaccination. In November 2021 a nationwide booster vaccination campaign started, giving every individual the opportunity to receive an extra dose of an mRNA vaccine, again starting with the most vulnerable persons and healthcare workers. In light of the sharp increase in Omicron cases in the Netherlands, preventive measures were (re-)enforced on December 19th 2021. People had to work from home if possible, shops, sport facilities and events had to close at 17o’clock, and face masks and physical distancing were mandatory in public places. When the number of hospitalized patients decreased and Omicron proved to put less pressure on the health care system than previous variants, measures were gradually lifted, with most measures removed at the end of February 2022.

### Vaccines

In the Netherlands, the initial vaccination campaign made use of all four vaccines approved for use by the European Medicine Agency (EMA) at that time, including two mRNA vaccines; BioNTech/Pfizer (BioNTech, Mainz, Germany & Pfizer, New York City, NY, USA) and Moderna (Cambridge, MA, USA); and two vector vaccines: AstraZeneca (Cambridge, UK) and Janssen (Johnson & Johnson, New Brunswick, NJ, USA). For all vaccinations, two doses were required to be regarded as fully vaccinated, with the exception of Janssen, for which one dose was sufficient. After reports of side effects caused by the AstraZeneca vaccine, it was decided to only offer this vaccine to individuals aged > 60 years. The administered vaccine depended on vaccine availability and year of birth. For this study, we assumed the type of vaccine used based year of birth. Individuals born in 1956–1960 were vaccinated with AstraZeneca; the birth cohorts of 1967, 1968, and 1976 received Janssen; all other age cohorts received Moderna or BioNTech/Pfizer with the exception of a minority of mostly younger individuals that could opt for a Janssen vaccine in June 2021, enabling them to rapidly obtain a Digital Corona Certificate.

### Study population

By law, Sanquin is the only organization allowed to collect and distribute blood components in the Netherlands. Around 400.000 Dutch donors make more than 700.000 donations a year at one of the 50 fixed or 87 mobile blood collection sites throughout the Netherlands [13]. To donate, donors must be aged between 18 and 79 years, be in good health, and pass the pre-donation health check. Prior to donation, donors had to complete a “corona check” (mandatory from March 2020-June 2022), answering questions about COVID-19 related symptoms. Following vaccination against SARS-CoV-2, donors were deferred for 7 days. Donors recovering from a recent SARS-CoV-2 infection were deferred until they were without symptoms for 14 consecutive days.

### Data collection

#### Study design

**Fig. 1:**
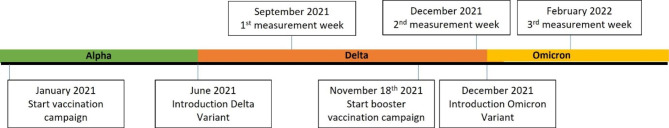
Timeline of measurement weeks, start of vaccination and booster campaign, and most dominant SARS-CoV-2 variant

In this cross-sectional study, SARS-CoV-2 antibody measurements were performed on samples of at least 2000 blood donations per week, collected during three separate weeks: September 13-16th 2021, November 30th - December 3rd 2021, and February 14-17th 2022 (Fig. 1). Samples were tested using three different SARS-CoV-2 antibody assays (see below). Samples were collected only from voluntary, non-remunerated, adult donors who provided written informed consent as part of routine donor selection and blood collection procedures. The study was reviewed and approved by the Ethics Advisory Council of Sanquin Blood Supply Foundation.

#### Sample selection

Donations were selected based on age, sex, and region of residence, to provide optimal coverage and representation of the population [14]. Specifically, the number of donations collected for every combination of the first three digits of the postal code of the donor’s residence, 10-year age group and sex was calculated for the period between January 1st 2019 and October 11th 2020. Only donations made in donation centers that are open at least once every two weeks were included to prevent large fluctuations in the number of donations from different areas. Without this correction, by chance a disproportionate share of donations could have been included from mobile locations that are only active a few times per year.

The number of desired donations (based on ideal representation of each group) for each combination of postal code, sex and age group was divided by the actual number of donations for each combination and the value obtained was used to make the selection of donations each day. A table containing these values for all donors was made in Excel for this purpose. Every morning, a list of donor numbers from donors who made a donation the previous day was inserted into the selection Excel file and a selection of 550 donations per day was made in a Monte-Carlo like fashion using the random number generator from Excel. Donors were enrolled if they were accepted for routine donation and gave permission for use of leftover samples for research. For the selection of donations at the February 2022 time point, younger donors (aged < 25) were oversampled, to further explore infection rates in this age group.

### Antibody assays

All samples were tested for RBD antibodies using the Wantai Ab ELISA (Wantai Biological Pharmacy Enterprise Co., Ltd., Beijing, China) [15]. The kits were obtained from the national stock of SARS-CoV-2 test kits. To enable the continued use of these test kits, despite their expiration in March 2021, each test week the test results from three 96-well plates were compared with the results of newly obtained kits, to assess potential changes in sensitivity or specificity. Samples showing seropositivity were additionally tested using the Elecsys® Anti-SARS-CoV-2 (Roche Diagnostics, Rotkreuz, Switzerland) assay for presence of NC antibodies; and the SARS-CoV-2 IgG II Quant assay (Abbott, Chicago, IL, USA) for quantification of RBD antibodies. The quantitative results were converted to the WHO International standard ‘Binding Antibody Units’ (BAU/mL) by multiplying the arbitrary units from the Abbott test results by 0.142, as described by the manufacturer. The Elecsys® Anti-SARS-CoV-2 assay enabled differentiation between infected individuals and persons who only received vaccination, since vaccinated individuals do not show an antibody response to the SARS-CoV-2 NC protein.

### Statistics

Donors were classified as “only vaccinated” if they had a positive test result in the Wantai test, while testing negative for anti-NC antibodies [16]. Donors testing positive for RBD and NC antibodies were considered previously infected (these donors might also be vaccinated, but there is no simple method for further differentiation). Donors showing no antibody response against either RBD nor NC were considered not vaccinated and not infected. Region was categorized into North (provinces of Groningen, Flevoland, Overijssel, Drenthe, Friesland), Mid-East (Gelderland), Mid-West (Utrecht, North Holland), South-East (North-Brabant, Limburg), and South-West (South Holland, Zeeland).

Data were processed and analyzed with R Studio, using the package *ggplot2* for generating graphs. In graphs, antibody titers were presented as the mean of log-transformed levels. Differences between groups were tested using Chi-square tests in case of proportions. Because of the skewed nature of antibody levels, we tested for normality using the Shapiro test. Because of a non-normal distribution of the data, we used the Mann-Whitney U test to test for differences between antibody titers, instead of a two-sample t-test, and we reported geometric means. Results from statistical testing procedures are reported as p-values, Hodges-Lehmann estimate and 95% confidence intervals (CI95%).

## Results

For each of the three time points over 2000 donors participated, with characteristics as reported in Table 1. Older donors were overrepresented as compared to younger donors, and the majority of participants lived in the south of the Netherlands. The proportion of “vaccine only” donors decreased between September 2021 and February 2022 from 75 to 64% (p < 0.001), as the amount of infected (and often also vaccinated) donors increased from 20 to 32% (p < 0.001). The proportion of donors that was neither vaccinated nor infected, without detectable antibodies, decreased from 5% in September 2021 to 2% in February 2022 (p < 0.001). Supplementary Fig. 1 displays the comparison of test results as obtained using the expired national stock kits, and fresh kit lots, showing excellent agreement.

**Table 1:** Population characteristics

	Sept 2021	Dec 2021	Feb 2022
**Donors tested**	2081	2121	2206
**Female (%)**	1007 (48)	989 (47)	990 (45)
**Age-group (%)**			
**18–25**	130 (6)	136 (6)	187 (9)
**26–35**	270 (13)	246 (12)	251 (11)
**36–45**	344 (17)	318 (15)	284 (13)
**46–55**	444 (21)	483 (23)	436 (20)
**56–65**	437 (21)	469 (22)	525 (24)
**66–75**	456 (22)	469 (22)	523 (24)
**Region (%)**			
**North**	480 (23)	495 (23)	505 (23)
**Mid-East**	223 (11)	286 (14)	271 (12)
**Mid-West**	391 (19)	414 (20)	410 (19)
**South-East**	501 (24)	473 (22)	506 (23)
**South-West**	485 (23)	453 (21)	514 (23)
**Antibody status (%)** ^*^			
**Only vaccinated**	1565 (75)	1603 (76)	1413 (64)
**Infected (+/- vaccinated)**	419 (20)	461 (22)	755 (32)
**Negative**	97 (5)	57 (3)	38 (2)

^*^Based on RBD and/or NC antibody response

**Fig. 2:**
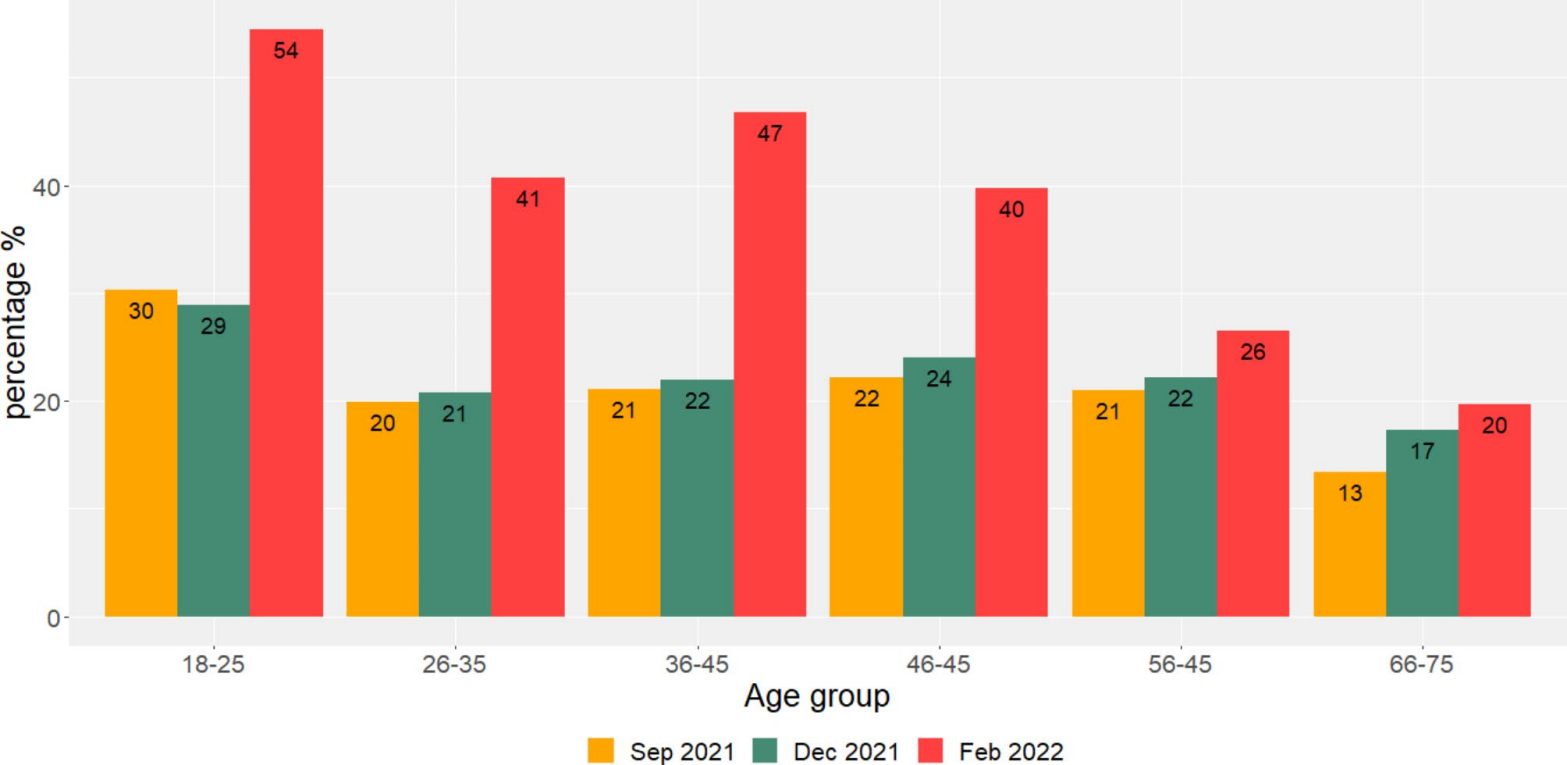
Percentage of donors with infection-acquired SARS-CoV-2 antibodies by age group and per cross-sectional cohort

**Fig. 3:**
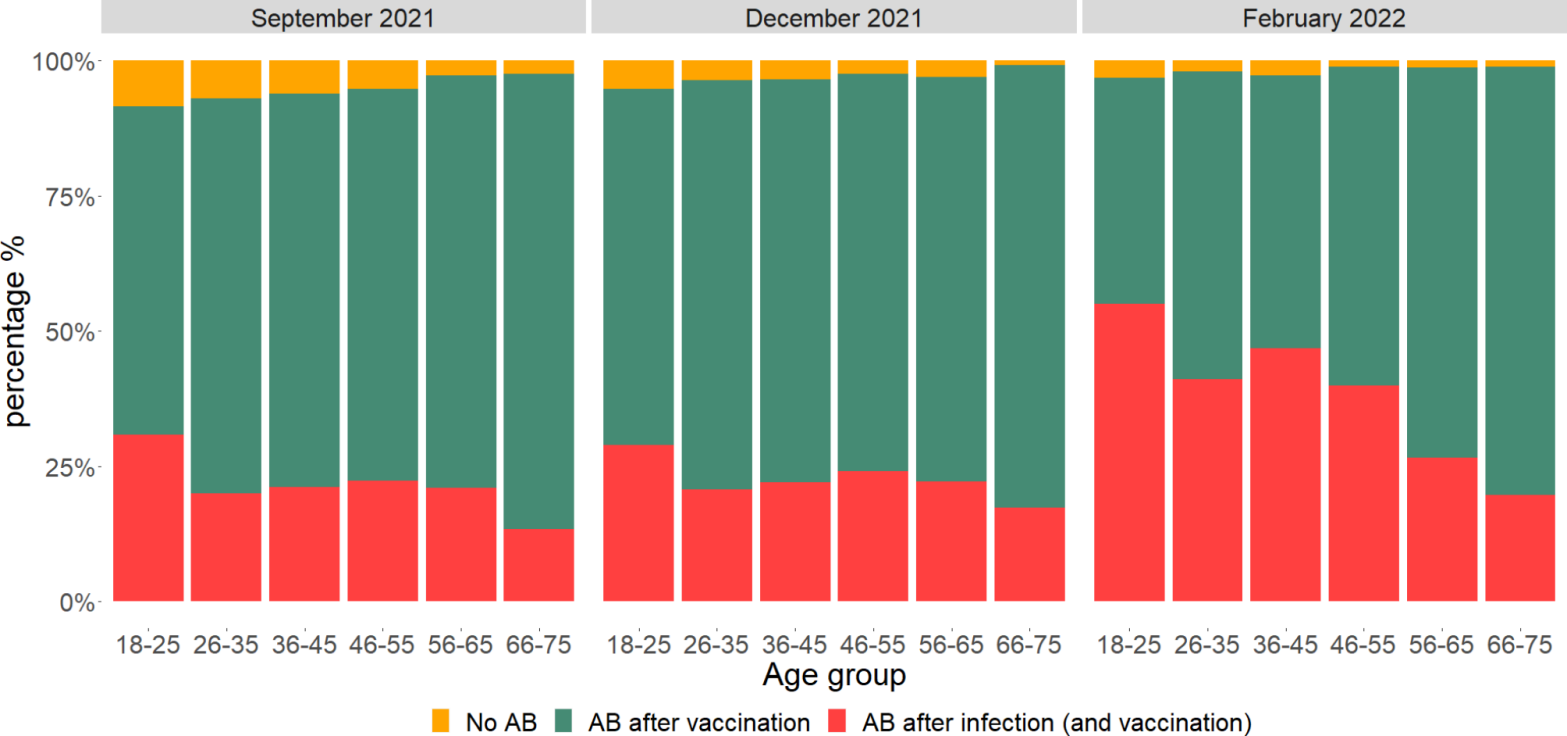
Percentage of donors with antibodies (AB) acquired after vaccination or infection (and vaccination) by age group

As depicted in Fig. 2, the proportion of infected donors increased over time in all age groups. In donors aged < 56 years the proportion of infected donors almost doubled from December 2021 to February 2022. The number of infections also increased in donors aged > 55, but not to the same extent as in younger donors. Regarding vaccine and infection-derived antibodies, the number of donors with no antibodies against SARS-CoV-2 decreased in each age-group over time, but was only significant in the age groups 18–25, 26–35, and 46–55. (Fig. 3). In February 2022, in the oldest age group (66–75 years) only 1% of donors had no antibodies against SARS-CoV-2, while in September 2021 this was 2.4% (Fig. 3). The majority of this age group was vaccinated and did not contract a SARS-CoV-2 infection (79%). In contrast, in the youngest age group (aged < 26), 55% of donors contracted SARS-CoV-2 infection, and 4% was not vaccinated nor infected in February 2022.

**Fig. 4:**
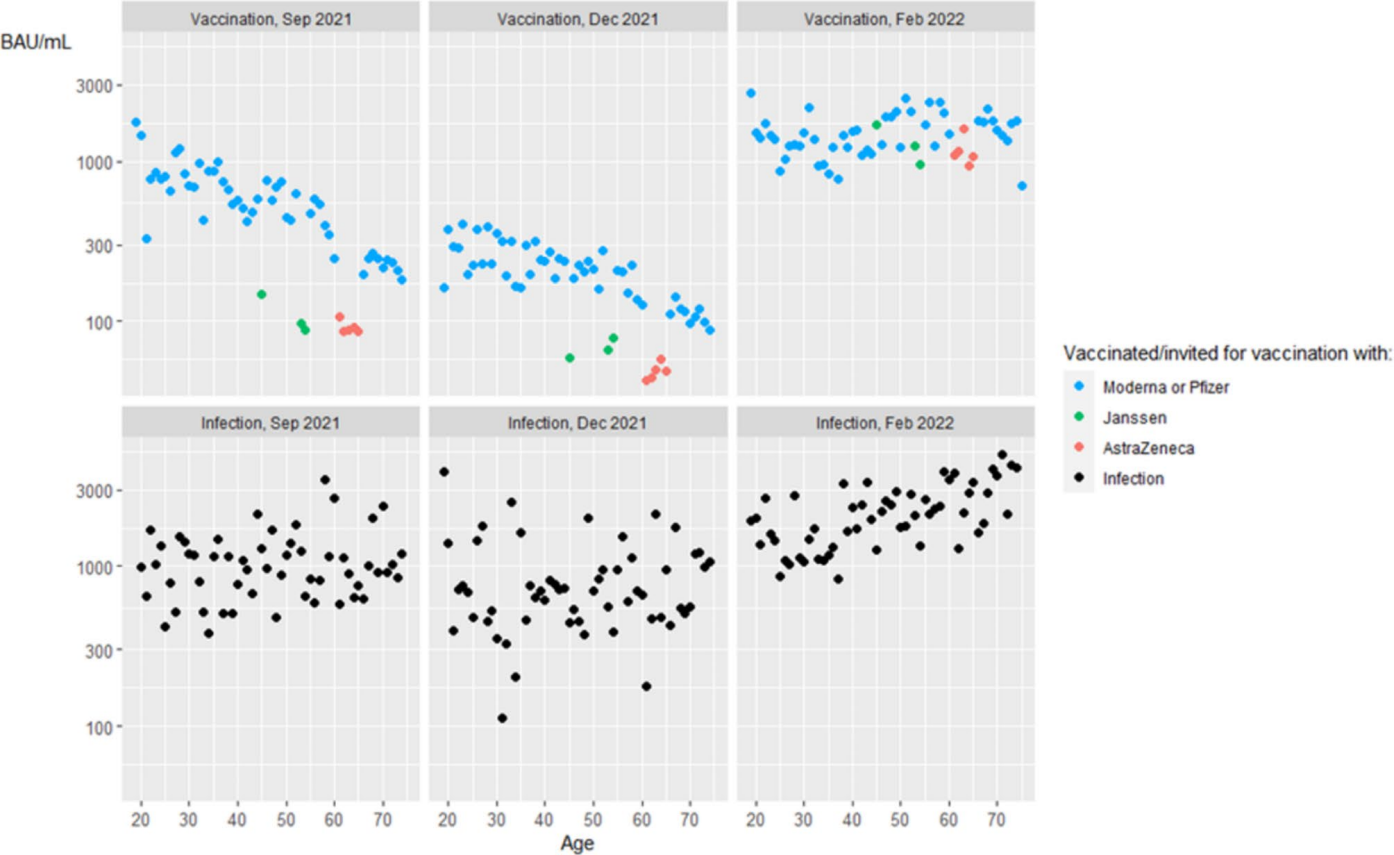
Booster vaccination increases mean antibody levels. Dots represent mean of log-transformed antibody levels by age-group in donors with antibodies acquired by Moderna or Pfizer (blue), Janssen (green) or AstraZeneca (red) vaccination, or infection (and vaccination) acquired antibodies (black)

The mean levels of vaccine-induced RBD antibodies, expressed in Binding Antibody Units (BAU) per mL, decreased between September-December 2021 in all age groups (Fig. 4, **upper row**, Table 2), with exception of the donors aged 46–55 vaccinated with Janssen. Donors vaccinated with either Janssen (p < 0.001) or AstraZeneca (p < 0.001) had significantly lower antibody levels as compared to those vaccinated with Pfizer or Moderna in September 2021. This remained true in December 2021 for both Janssen (p < 0.001) and AstraZeneca (p < 0.001). After the booster vaccination campaign, which started November 2021, a significant increase in antibody levels was observed in all age groups, irrespective of the vaccine that was used initially (Table 2). Donors who initially received the Janssen or AstraZeneca vaccine, and who were boostered with an mRNA vaccine, showed a quantitative catch-up effect and reached levels comparable to the donors who were initially vaccinated using an mRNA vaccine.

**Table 2:** Geometric means of antibody titer (BAU/mL) stratified by age group and measurement week

	September 2021	December 2021	February 2022	September vs. December	December vs. February
	Geometric mean of antibody titer (BAU/mL)			Hodges-Lehmann estimate [95% CI]^&^	
Pfizer or Moderna					
18–25	780.5	295.6	1397.8	834.0 [346.2,1149.3]***	-1064.0 [-820.2, -1288.5]***
26–35	797.1	254.0	1174.1	681.0 [518.9, 852.9]***	-981.2 [-831.9, -1130.9]***
36–45	582.4	243.2	1178.7	456.6 [368.4, 548.8]***	-1001.5 [-827.7, -1215.1]***
46–55	572.9	212.0	1766.7	442.1 [362.6, 519.8]***	-1793.8 [-1564.3, -2039.0]***
56–65	405.6	164.7	1825.1	219.2 [165.6, 278.6]***	-1848.8 [-1547.5, -2232.3]***
66–75	219.7	108.3	1667.1	98.2 [80.9, 116.5]***	-1633.3 [-1490.6, -1761.9]***
Total	444.2	181.6	1552.9	239.8 [206.5, 277,3]***	-1433.3 [-1355.1, -1510.1]***
**Janssen^**					
36–45	147.5	59.4	1682.3	55.9 [2.8, 272.8]*	-1800.2 [-947.0, -2295.4]***
46–55	92.7	71.9	1107.3	7.4 [-14.2, 28.8]^ns^	-884.1 [-743.7, -1316.1]***
Total	103.3	68.0	1213.44	14.4 [-1.9, 36.5]^ns^	-1026.6 [-802.3, -1454,6]***
**AstraZeneca** ^**#**^					
56–65	90.3	48.5	1155.0	35.6 [21.8, 51.8]***	-1165.9 [-1009.2, -1272.2]***
**Previous infection**					
18–25	1019.7	689.7	1659.6	310.7 [-101.0, 893.1]^ns^	-889.6 [-411.2, -1448.4]***
26–35	890.6	549.5	1239.4	274.6 [-64.2, 779.9]^ns^	-595.7 [-238.6, -1005.6]**
36–45	910.1	650.9	1781.0	363.5 [-20.7, 864.2]^ns^	-984.3 [-519.2, -1490.2]***
46–55	1016.0	679.2	2162.1	417.7 [106.7, 771.2]**	-1432.3 [-1025.8, -1982.2]***
56–65	1027.7	769.8	2625.2	142.8 [-128.4, 443.6]^ns^	-1784.3 [-1333.4, -2334.0]***
66–75	1099.7	861.8	2985.2	150.4 [-185.4, 503.1]^ns^	-1712.0 [-1090.0, -2595.9]***
Total	994.2	708.2	2022.7	263.2 [126.8, 416,5]***	-1219.4 [-1015.0, -1432.4]***

^birth cohorts 1967, 1968, and 1976

^#^ birth cohorts 1956–1960

^&^ Tested with Mann-Withney U test

* = p < 0.05, ** = p < 0.01, *** = p < 0.001 ^ns^ = not significant

Donors who experienced SARS-CoV-2 infection, with or without additional vaccination, showed higher antibody levels as compared to donors that were only vaccinated, especially in older age groups (Fig. 4, bottom row) in September 2021 (p < 0.001) and December 2021 (p < 0.001). After the booster vaccination campaign, previously infected donors show higher antibody levels than boostered donors that were vaccinated only (p < 0.001).

## Discussion

This study aimed to explore the extent of changes in mean SARS-CoV-2 antibody levels across age groups, the impact of the introduction of Omicron strains on infection rates, and the effect of a booster vaccination campaign on antibody levels. We observed a large increase of donors infected with SARS-CoV-2 between December 2021 and February 2022, especially in the younger age groups. The proportion of donors without antibodies against SARS-CoV-2 only slightly decreased, suggesting re- and breakthrough infections in antibody positive donors in the period between December 2021 and February 2022. Each age group showed decreased mean vaccine-induced antibodies, and age groups that were vaccinated with the Janssen or AstraZeneca vaccine showed lower antibody levels compared to the mRNA vaccines prior to booster vaccination. The booster campaign restored decreased antibody titers, as seen by the significantly higher antibody levels in February 2022, fortunately bringing antibody levels of donors vaccinated with Janssen or AstraZeneca to comparable levels as those originally vaccinated with an mRNA vaccine.

The significant increase in infections between December 2021 and February 2022 can be explained in two ways. First, starting January 2022, most infection preventive measures were gradually lifted, causing more contacts between individuals and thus more infections. Second, protection against infection is reduced for the Omicron variant, even in boostered individuals [17]. Since the vaccine coverage at the time was lower in younger age groups - below 75% in individuals aged < 40 and below 85% in individuals aged < 50 years old - is it not surprising that the number of infections almost doubled in donors younger than 55 years old between December 2021 and February 2022. Of note is that the number of donors that was never infected nor vaccinated decreased from 3 to 2% between December 2021 and February 2022, indicating that only few “antibody-naïve” donors were infected in this period. This suggests that the increase in NC-positive donors is caused by breakthrough infections in antibody positive donors in the period between September 2021 and February 2022, which is not unlikely since Omicron is able to evade vaccine and previous infection induced immunity [18].

A reduction of levels of vaccine induced antibodies in donors without prior infection was observed in almost all age groups between the first time points of the study. Donors vaccinated with AstraZeneca or Janssen showed lower antibody levels compared to those vaccinated with an mRNA vaccine. However, after the booster campaign, during which all vaccinated individuals received an mRNA vaccine, antibody levels were restored to the same level as in boostered donors previously vaccinated with a mRNA vaccine. Future studies should show booster effectiveness in preventing (re-) infection and hospitalization by infections caused by Omicron variants and new SARS-CoV-2 variants of concern.

A major strength of this study is that it captures the impact of Delta and Omicron infection rates and, additionally, shows the effect of the booster vaccination on antibody levels. To our knowledge, this is the first national study using a blood donor population for this purpose. This study shows the potential of using blood donors for infectious disease monitoring. Since most blood donors donate on a regular basis, donors can be followed-up easily. Additionally, because informed consent procedures are incorporated in the blood donation process, no additional research logistics need to be implemented.

This study has limitations. First, we have no information on the vaccination status of the participants, including when they received vaccination, other than our antibody testing results. We assumed the received vaccine based on the year of birth. Second, though sensitivity of the Wantai Ab assay is high, some vaccinated donors might be missed because of a false negative result, and were therefore not tested with additional assays. Additionally, waning of NC antibodies and sero-reversion of donors could cause an underestimation of infected donors[19]. Third, donors must be healthy to donate. Donors having had a SARS-CoV-2 infection must be symptom-free for 14 days to be allowed to donate. Even then, donors might not feel healthy enough to donate. This could lead to an underestimation of infected donors. This applies in particular to the February time point, when the number of infections caused by Omicron was very high. Lastly, the estimation of the proportion of vaccinated and infected donors is not necessarily representative for the general Dutch population. This can be attributed to the healthy donor effect (HDE), a selection bias that favors the healthy part of the population that is allowed to donate blood.

In conclusion, the introduction of Omicron variants in the Netherlands led to a substantial increase in donors with infection induced antibodies, especially among younger donors. A booster vaccination campaign corrected decreased antibody levels in all age-groups. Donors initially vaccinated with AstraZeneca or Janssen showed boostered antibody levels similar to boostered donors initially vaccinated with a mRNA vaccine.

## Electronic supplementary material

Below is the link to the electronic supplementary material.

Supplementary Material 1

## Data Availability

The datasets used and/or analyzed during the current study are available from the corresponding author on reasonable request.
