# Supplementary material for: Booster vaccinations and Omicron: the effects on SARS-CoV-2 antibodies in Dutch blood donors
Source: BMC Infect Dis. 2023 Jul 12;23:464. doi: 10.1186/s12879-023-08448-w (PMC10339593; doi:10.1186/s12879-023-08448-w)
Supplement: Supplementary file 1 — Supplementary Material 1 [file 12879_2023_8448_MOESM1_ESM.docx]

## Supplementary Figure 1.


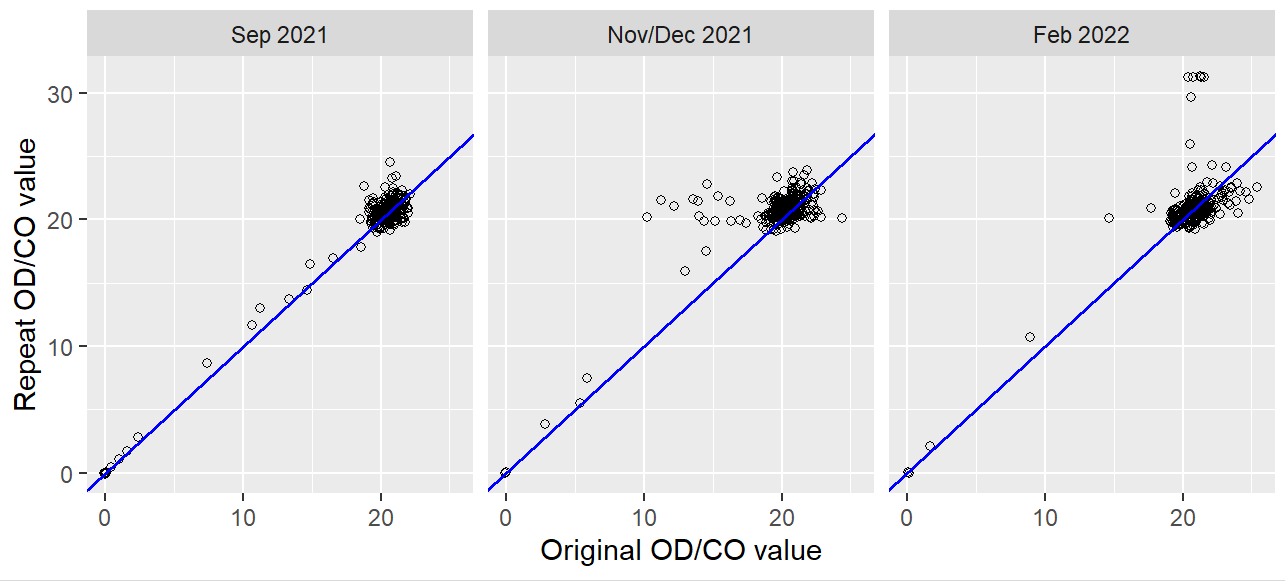


**Supplementary Figure 1. Results of repeat testing of samples with an OD/CO ratio>0.25 in the Wantai assay using either the kit lot used for initial screening or a recently produced kit lot. The solid line shows perfect agreement (y=x).**
